# Immunological effects and activity of multiple doses of zolbetuximab in combination with zoledronic acid and interleukin-2 in a phase 1 study in patients with advanced gastric and gastroesophageal junction cancer

**DOI:** 10.1007/s00432-022-04459-3

**Published:** 2023-01-06

**Authors:** Florian Lordick, Peter Thuss-Patience, Michael Bitzer, Daniel Maurus, Ugur Sahin, Özlem Türeci

**Affiliations:** 1 Department of Oncology, Gastroenterology, Hepatology, and Pulmonology, Comprehensive Cancer Center Central Germany (CCCG), University of Leipzig Medical Center, Liebigstraße 22, 04103 Leipzig, Germany; 2 Department of Hematology, Oncology and Tumor Immunology, Charité-University Medicine Berlin, Campus Virchow-Klinikum, Augustenburger Platz 1, 13353 Berlin, Germany; 3 Department of Internal Medicine I, University Hospital, Eberhard Karls University Tuebingen, Otfried-Müller-Straße 10, 72076 Tübingen, Germany; 4 Ganymed Pharmaceuticals GmbH (Formerly Ganymed Pharmaceuticals AG), Mainz, Germany; 5 Translational Oncology, University Medical Center, Johannes Gutenberg University Mainz, Mainz, Germany; 6 University Medical Center, Johannes Gutenberg University Mainz, Freiligrathstraße 12, 55131 Mainz, Germany; 7 Biopharmaceutical New Technologies (BioNTech) Corporation, An der Goldgrube 12, 55131 Mainz, Germany; 8 Present Address: Ci3-Cluster of Individualized Immune Intervention, Mainz, Germany

**Keywords:** Gastric cancer, Esophagogastric junction cancer, Zolbetuximab, Interleukin-2, Claudin 18.2, Antibody-dependent cell-mediated cytotoxicity

## Abstract

**Purpose:**

Zolbetuximab (IMAB362) is engineered to induce antibody-dependent cell-mediated cytotoxicity (ADCC) and complement-dependent cytotoxicity. We evaluated ADCC activity and the impact of the immune-modulating drugs zoledronic acid (ZA) and interleukin-2 (IL-2) as co-treatment with zolbetuximab on relevant immune cell populations and ADCC lysis activity.

**Methods:**

This phase 1, multicenter, open-label study investigated the immunological effects and activity, safety, tolerability, and antitumor activity of multiple doses of zolbetuximab alone (*n* = 5) or in combination with ZA (*n* = 7) or with ZA plus two different dose levels of IL-2 (low dose: 1 million international units [mIU] [*n* = 9]; intermediate dose: 3 mIU [*n* = 7]) in pretreated patients with advanced gastric and gastroesophageal junction (G/GEJ) adenocarcinoma.

**Results:**

Twenty-eight patients with previously treated advanced G/GEJ adenocarcinoma that was CLDN18.2-expressing were enrolled into four treatment arms. Treatment with zolbetuximab + ZA + IL-2 induced short-lived expansion and activation of ADCC-mediating cell populations, namely γ9δ2 T cells and natural killer cells, within 2 days after administration; this effect was more pronounced with intermediate-dose IL-2. Expansion and activation of regulatory T cells treated with either IL2 dose was moderate and short-lived. Strong ADCC activity was observed with zolbetuximab alone. Short-lived ADCC activity was observed in several patients treated with ZA + intermediate-dose IL-2, but not lower-dose IL-2. In the clinical efficacy population, the best confirmed response was stable disease (*n* = 11/19; 58%).

**Conclusions:**

Zolbetuximab mediates proficient ADCC in patients with pretreated advanced G/GEJ cancers. Co-treatment with ZA + IL-2 did not further improve this effect.

**Trial registration:** NCT01671774

**Supplementary Information:**

The online version contains supplementary material available at 10.1007/s00432-022-04459-3.

## Introduction

Gastric cancer is the fifth most common cancer and the fourth most common cause of cancer death globally (Sung et al. 2021). As gastric adenocarcinomas are typically detected at an advanced stage, treatment outcomes are dismal (Bilici 2014; Kanagavel et al. 2015). Chemotherapy is the mainstay of treatment for advanced tumors and a platinum/fluoropyrimidine-based doublet is administered in most cases (Lordick et al. 2022; Wagner et al. 2017). The only widely used molecularly targeted agents currently approved in gastroesophageal cancer are trastuzumab in first-line therapy for tumors that overexpress the human epidermal growth factor receptor 2 (HER2) (Bang et al. 2010) and ramucirumab in second-line therapy (Fuchs et al. 2014; Wilke et al. 2014). Pembrolizumab, a programmed cell death protein-1 (PD-1) inhibitor, is approved in the United States in combination with chemotherapy and trastuzumab as first-line therapy for treatment of HER2 + disease (Keytruda [pembrolizumab], US prescribing information, 2022). Nivolumab is approved in the United State in combination with chemotherapy as a first-line therapy for advanced gastric cancer (Opdivo [nivolumab], US prescribing information, 2022). In Europe, the use of immune checkpoint inhibitors is largely restricted to patients with PD-L1–positive gastric, gastroesophageal, or esophageal tumors, particularly those with a combined positive score of ≥ 5 (European Medicines Agency, Keytruda [pembrolizumab], 2022a; European Medicines Agency, Opdivo [nivolumab], 2022b). Despite numerous molecular targeted therapies, immunotherapies, and novel chemotherapies under investigation, there is an urgent need for treatments that improve survival in advanced gastric and gastroesophageal junction (G/GEJ) adenocarcinomas (Nie et al. 2020).

Zolbetuximab (previously known as IMAB362) is a first-in-development monoclonal IgG1 antibody against splice variant 2 of claudin 18 (CLDN18.2). Claudin family members are tight junction proteins, which establish cell polarity and regulate cellular permeability in epithelial cells (Rendon-Huerta et al. 2013; Singh et al. 2010; Tureci et al. 2011). In normal tissues, CLDN18.2 expression is restricted to the differentiated epithelial cells of the gastric mucosa and this lineage-specific expression is maintained even after malignant transformation (Sahin et al. 2008). CLDN18.2 is sequestered in the normal gastric epithelia to the apical and lateral membrane regions, whereas in gastric cancer, this sequestration may be lost due to perturbations in cell polarity making CLDN18.2 better accessible to immune effectors (Rendon-Huerta et al. 2013; Sahin et al. 2008). Furthermore, aberrant activation of CLDN18.2 expression is detected in ovarian, esophageal, and pancreatic malignancies (Sahin et al. 2008), suggesting that CLDN18.2 may serve as a therapeutic target for multiple tumor types. As a single agent, zolbetuximab has demonstrated safety/tolerability and antitumor activity in early phase studies in patients with gastric, GEJ, or esophageal adenocarcinoma (Sahin et al. 2018; Tureci et al. 2019b). In a randomized phase 2 study (FAST, NCT01630083), patients with CLDN18.2-positive advanced G/GEJ cancer treated with zolbetuximab + epirubicin, oxaliplatin, and capecitabine (EOX) showed prolonged progression-free survival (PFS) and overall survival (OS) and improved patient-reported outcomes compared with patients treated with EOX alone (Sahin et al. 2021).

Zolbetuximab exerts its antitumor activity by antigen-specific activation of immune effector mechanisms and induces both antibody-dependent cell-mediated cytotoxicity (ADCC) and complement-dependent cytotoxicity (CDC) (Singh et al. 2017; Tureci et al. 2019a). ADCC describes the capability of cell-bound zolbetuximab to mediate killing by engaging Fc receptor III-bearing (FcγRIII, CD16) immune effector cells, namely natural killer (NK) cells and gamma/delta (γδ) T cells. γδ T cells are considered particularly potent mediators of ADCC, but in general constitute a rather small population of T cells.

We hypothesized that compounds that activate and expand γδ lymphocytes could augment zolbetuximab-mediated ADCC. The bisphosphonate zoledronic acid (ZA) is known to induce diverse immunological effects, including activation of cytolytic γ9δ2 T cells and NK cells (Lang et al. 2011; Mueller et al. 2013). The γδ lymphocyte subset recognizes phosphoantigens, such as isopentenyl pyrophosphate (IPP), which are produced in mammalian cells by the mevalonate pathway. ZA causes intracellular accumulation of IPP by inhibiting the IPP-metabolizing enzyme in the mevalonate pathway. In exploratory clinical trials, intravenous (IV) ZA (4 mg) + subcutaneous (SC) IL-2 (0.6 million international units [mIU]) administered every 3 weeks (Q3W) resulted in a sustained increase in peripheral γ9δ2 T cells and clinical responses in patients with metastatic prostate cancer (Dieli et al. 2007). In contrast, IV ZA (4 mg; Day 1 of each 28-day cycle) and increasing doses of IL-2 (1 mIU to 7 mIU; Days 1–5 on Weeks 1–3 of each 28-day cycle) did not yield sustained increases in γ9δ2 T cells or clinical responses in patients with refractory renal cell carcinoma (Lang et al. 2011).

We evaluated zolbetuximab in combination with ZA and IL-2 in patients with advanced or metastatic G/GEJ adenocarcinoma to determine activation and kinetic profiles of immune cell populations induced by ZA with or without IL-2.

## Materials and methods

### Study design and participants

Participants in this phase 1, multicenter, open-label, exploratory study (NCT01671774) of zolbetuximab in combination with ZA and IL-2 were adults (aged ≥ 18 years) with refractory or recurrent histologically confirmed, advanced adenocarcinomas of the stomach, esophagus, or GEJ who had an Eastern Cooperative Oncology Group (ECOG) performance status ≤ 1. The study was conducted across nine sites in Germany and Latvia between October 2012 and October 2014. All patients underwent screening for CLDN18.2 expression; only patients with tumor samples with immunohistochemically confirmed higher and strong CLDN18.2 expression (3 + staining intensity, or 2 + staining intensity in ≥ 40% of tumor cells) were eligible. A complete list of inclusion and exclusion criteria is presented in Supplementary Table 1*.* The study protocol and informed consent forms were reviewed and approved by the Independent Ethics Committee(s) (IEC) at each participating investigational center. The study was conducted in accordance with the ethical principles of the Declaration of Helsinki, Good Clinical Practice guidelines, and the requirements of public registration of clinical trials. Written informed consent was obtained from each patient at the time of enrollment.

The primary study objectives were to explore the pharmacodynamics, including activation and kinetic profiles, of various immune cell populations (i.e., γ9δ2 T cells, NK cells, regulatory T [T_reg_] cells, B cells, monocytes) induced by ZA, with or without IL-2, in relation to zolbetuximab-induced activity, and to determine the safety/tolerability profile of zolbetuximab in combination with ZA with or without IL-2. Antitumor activity was a secondary objective.

### Treatment administration

Patients were randomly assigned to one of four treatment arms: Arm 1: zolbetuximab Q3W + ZA (4 mg); Arm 2: zolbetuximab Q3W + ZA (4 mg) + low-dose IL-2 (1 mIU); Arm 3: zolbetuximab Q3W + ZA (4 mg) + intermediate-dose IL-2 (3 mIU); Arm 4: zolbetuximab Q3W. In all treatment arms, zolbetuximab was administered as a 2-h IV infusion at a dose of 800 mg/m^2^ on Day 1 of Cycle 1, and at a dose of 600 mg/m^2^ on Day 1 of each subsequent cycle. Treatment with zolbetuximab was continued until the occurrence of disease progression, unacceptable toxicity, or patient withdrawal of consent. In Arms 1, 2, and 3, zolbetuximab was administered prior to the 15-min IV infusion of ZA on Day 1 of Cycles 1 and 3. In Arms 2 and 3, IL-2 1 mIU and 3 mIU, respectively, was administered as an SC injection on Days 1, 2, and 3 of Cycles 1 and 3. No dose reductions for zolbetuximab were permitted; however, a dose delay of up to 2 days was allowed. Dose delays of up to 2 days were also allowed for ZA and IL-2. Further details regarding IL-2 dose interruptions and reductions are provided in Supplementary Table 2.

### Immune response evaluation

**Fig. 1:**
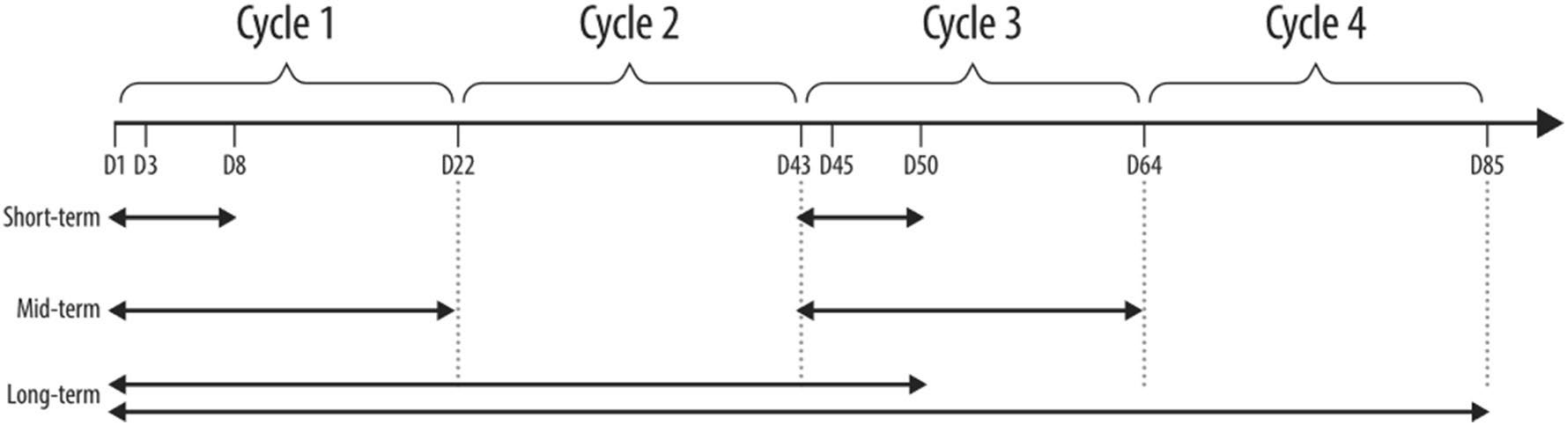
Definition of time frames for the evaluation of immunomodulatory effect of ZA/IL-2 in combination with zolbetuximab. *D* Day

Analyses of immune cell profile and kinetics were performed before and during treatment with zolbetuximab in combination with ZA with/without IL-2. Changes in immunological profile over time and induction of ADCC by zolbetuximab across treatment arms were analyzed. The immunomodulatory effects of ZA/IL-2 in combination with zolbetuximab during treatment were assessed by examining short-term, mid-term, and long-term changes (Fig. 1) in immune cell populations, phenotypes, or activation markers. Peripheral blood mononuclear cell (PBMC) samples were obtained for pre-treatment analyses on Day 1 (the same day at which treatment was initiated). Pre-treatment Day 1 samples were compared to Day 3 and/or Day 8 samples. Additionally, short-term impact of ZA/IL-2 was addressed in Cycle 3 by comparing Day 43 samples to Day 45 and/or Day 50 samples. Mid-term changes were defined as changes throughout each of the ZA/IL-2 treatment cycles, such as changes between Day 1 and Day 22, and between Day 43 and Day 64. Long-term changes were evaluated by comparing Day 1 to Day 50, and Day 1 to Day 65 and/or Day 85 if samples for at least three patients for at least two study arms were available.

Immune phenotyping of PBMCs was performed by the central lab, TRON gGmbH, according to TRON working instructions. The number of T cell, γδ T cells, NK cells, B cells, monocytes and T_reg_ cells were analyzed and calculated as the percentage of the PBMC sample using flow cytometry (BD FACSCanto II, BD Biosciences, Erembodegem, Belgium) in three panels. The number of cells per sample stained in each of the three panels was aimed to be 1 × 10^6^, if enough viable cells were available. If less than 3 × 10^6^ viable PBMCs were available (1 × 10^6^ per panel), the maximum number of viable cells for each of the patient visits was used per test. No less than 0.5 × 10^6^ PBMCs were stained in each of the three panels. Titration and interday and intraday variability were determined by flow cytometry for each of the subpopulations and activation/memory markers. Immune phenotyping results were interpreted according to interday variability of the respective assay, either quantitatively (relative CV < 25%) or qualitatively (relative CV ≥ 25%).

Whether the observed immunological profiles had an effect on defined modes of action of zolbetuximab was evaluated by harvesting and testing ex vivo PBMCs as a source for ADCC at Day 1 before the first ZA or ZA + IL-2 treatment; Day 43 before the second ZA or ZA + IL-2 treatment; and posttreatment on Days 8, 22, 50, 64, and 85. The ADCC assay and measurements were developed and performed by Ganymed and are described in detail elsewhere (Tureci et al. 2019a). Briefly, PBMCs, as ADCC effector cells (E), were isolated from healthy donors (Transfusionszentrale, Mainz). NUGC4 human gastric cancer cell lines, as target cells (T), were prepared by transient transfection with Anti-Reverse-capped (ARCA, Ambion) luciferase RNA (TRON GmbH, Mainz, Germany) to allow detection of cell lysis. Target cells were cultured before the addition of antibodies and effector cells; zolbetuximab and control isotype antibodies were serially diluted at concentrations ranging from 0.26 ng/mL to 200 µg/mL. PBMCs were added at an E:T ratio of 40:1 and assay plates were incubated for 24 h at 37°. D-luciferin (BD Biosciences) was added and the reaction incubated for 80 min at room temperature in the dark. Cells were lysed and assayed for luminescence.

### Clinical evaluation

Safety and tolerability were assessed by monitoring treatment-emergent adverse events (AEs), clinical laboratory test results, vital signs, electrocardiogram measurements, left ventricular ejection fraction results, ECOG performance status, and physical examination results. Adverse events were graded by their severity (CTCAE v4.03) and categorized according to their seriousness and relationship to the study treatment.

The presence of antidrug antibodies (ADAs) was evaluated in blood samples collected on Day 1 of Cycle 1 prior to the first zolbetuximab administration and during the end-of-treatment assessment. For patients who continued zolbetuximab treatment after the immunomodulation phase, blood samples for ADA evaluation were also collected at Week 13 prior to zolbetuximab administration.

Tumor response was assessed by contrast-enhanced computed tomography (CT) or magnetic resonance imaging and was evaluated using Response Evaluation Criteria in Solid Tumors v1.1 criteria. Clinical response, including objective response rate (ORR) and duration of response, was assessed.

### Statistical analysis

As an exploratory study, no formal sample size calculations were performed. The safety population included all enrolled patients who received ≥ 1 dose of any study medication. The per-protocol set was defined as all enrolled patients who had no protocol violations and who had met one of the following criteria: (1) completion of ≥ 2 cycles of study medication with evaluable blood samples for FACS or ADCC at baseline and ≥ 1 postbaseline time point; (2) completion of one cycle of study medication with evaluable pretreatment (Day 1 Cycle 1) blood samples and complete evaluable blood samples at Cycle 1 for FACS (Days 3 and 8) and ADCC (Day 8). The clinical efficacy population included all enrolled patients who received ≥ 1 dose of any study medication and who also had at least one postbaseline CT scan.

Continuous variables were summarized by frequency, mean ± standard deviation, median, interquartile range, and minimum and maximum values. Categorical variables were summarized by number and percentage of patients in each category. Arm-wise comparison of immune cell phenotyping was only carried out when values of at least three patients were available for each arm and the coefficient of variation for the respective cell type was lower than 25%. This resulted in Arms 2 and 3 being combined for some analyses.

In order to normalize temporal change rates, frequencies of immune cells were calculated as a ratio of the posttreatment to pretreatment percentage of a defined cell subpopulation per patient. The median change factor, with the upper (Q1) and lower quartile (Q3), were calculated for each treatment arm. In addition to the relative change rates to baseline, the actual frequency differences (Δ%) were calculated to verify if high relative change rates are reflected by a significant change. The analysis of actual frequency differences is only mentioned in the results section if analysis of relative change rates to baseline would otherwise convey a misleading impression, e.g., if obtained data was close to the detection limit of the assay. To improve interpretation in ambiguous cases, total cell numbers were determined by back calculation in a non-validated analysis to confirm that relative and absolute changes in cell frequency are reflected in the respective subpopulation. Total lymphocyte and living cell numbers of patients were obtained for posttreatment Days 22, 43, 64, and 85 from the complete blood count. These non-validated analyses are only presented if they were helpful for the interpretation of cell frequency change.

## Results

### Baseline patient characteristic

A total of 28 patients received zolbetuximab and were included in the safety population; of these patients, 21 were included in the pharmacodynamic analyses and 19 were included in the clinical efficacy population. The majority of patients were male with a median age of 56.5 years (Table 1). Metastatic disease was present in 27 (96.4%) patients. All patients in the clinical efficacy population had received prior chemotherapy. Most had undergone prior surgery (*n* = 15/19; 78.9%) or had received prior adjuvant/neoadjuvant chemotherapy (*n* = 13/19; 68.4%). The number of prior lines of therapy was not collected.

**Table 1:** Demographic and baseline characteristics in the safety population

Parameters	Arm 1 zolbetuximab + ZA (*n* = 7)	Arm 2 zolbetuximab + ZA + IL-2 (1 mIU) (*n* = 9)	Arm 3 zolbetuximab + ZA + IL-2 (3 mIU) (*n* = 7)	Arm 4 zolbetuximab (*n* = 5)	Total (*N* = 28)
Median age, years (range)	48 (42–63)	58 (41–62)	54 (27–75)	70 (56–72)	56.5 (27–75)
Sex					
Male	7 (100.0)	7 (77.8)	4 (57.1)	3 (60.0)	21 (75.0)
Female	0	2 (22.2)	3 (42.9)	2 (40.0)	7 (25.0)
Median number of months since diagnosis (range)	17.5 (7–41.5)	11.7 (4.7–31.6)	21.6 (6.9–57.5)	30.8 (9.2–52.8)	16.6 (4.7–57.5)
Location of tumor^a^					
Esophagus	2 (28.6)	2 (22.2)	0	1 (20)	5 (18.5)
GEJ	2 (28.6)	4 (44.4)	2 (33.3)	3 (60)	11 (40.7)
Stomach	3 (42.9)	3 (33.3)	4 (66.7)	1 (20)	11 (40.7)
Type of tumor					
Diffuse	2 (28.6)	3 (33.3)	3 (42.9)	2 (40)	10 (35.7)
Intestinal	5 (71.4)	5 (55.6)	2 (28.6)	3 (60)	15 (53.6)
Mixed	0	0	1 (14.3)	0	1 (3.6)
Unknown	0	1 (11.1)	1 (14.3)	0	2 (7.1)
Median number of metastatic sites (range)	4 (2–6)	2 (1–5)	3 (1–5)	3 (2–5)	3 (1–6)
Maximum CLDN18.2 intensity					
2 +	0	2 (22.2)	3 (42.9)	1 (20)	6 (21.4)
3 +	7 (100)	7 (77.8)	4 (57.1)	4 (80)	22 (78.6)
Median (range) percentage of CLDN18.2-stained tumor cells^b^	80 (1–90)	60 (15–99)	55 (3–90)	75 (4–90)	65 (1–99)

Data presented as n (%) unless otherwise indicated

*CLDN18.2* claudin 18.2, *GEJ* gastroesophageal junction, *IL-2* interleukin 2, *IU* international units, *ZA* zoledronic acid

^a^Tumor location was known for six of seven patients in Arm 3

^b^Includes 2 + and 3 + staining intensities, and CLDN18.2 + tumor cells

### Phenotyping of blood immune cell populations

Relative immune cell frequencies across treatment arms are shown in Table 2. Following treatment with zolbetuximab + ZA + IL-2 (low-dose and intermediate-dose arms combined), a ≥ 3.0-fold increase in the γ9δ2 T-cell population was observed versus treatment with zolbetuximab alone. Zolbetuximab + ZA + IL-2 had little effect on T-cell and B-cell monocyte frequencies, and a moderate effect (< 0.8-fold and ≥ 0.5-fold) on NK cells, by Day 45. Compared with zolbetuximab alone, increased CD69^+^-activated γ9δ2 T cells and NK cells were observed after both cycles with ZA + IL-2 (≥ 3.0-fold by Day 45). Minor changes were observed in CD69 + -activated T cells (> 1.2-fold and < 2.0-fold by Day 45) and B cells (< 0.8-fold and ≥ 0.5-fold by Day 45). A brief expansion of CD69^+^-activated B cells was observed following zolbetuximab + ZA + IL-2 treatment during Cycle 1 (≥ 3.0-fold by Day 3), but not during Cycle 3 (< 0.8-fold and ≥ 0.5-fold by Day 45). Increases from baseline in immune cell subpopulations in patients treated with zolbetuximab + ZA were similar for CD69^+^-activated γ9δ2 T cells (≥ 3.0-fold) and CD69^+^ T cells (≥ 2.0-fold and < threefold) but were less pronounced for CD69^+^-activated NK and B cells.

**Table 2:** Relative immune cell frequencies across treatment arms

Cell type	Arm 1 vs. Arm 4				Arms 2 + 3 vs. Arm 4				IL-2 dose-dependency
	Cycle 1		Cycle 3		Cycle 1		Cycle 3		
	D3	D8	D45	D50	D3	D8	D45	D50	
*Cell lineages*									
γ9δ2 T cells	−	=	− −	− −	−	+ + +	+ +	=	Yes
T cells	=	+	=	=	=	=	=	=	No
NK cells	=	−	=	+	=	=	−	=	No
B cells	+	=	+	=	=	=	=	=	No
Monocytes	=	=	=	+	=	=	=	=	No
*Activation*									
CD69^+^ γ9δ2 T cells	+ + +	+ + +	+ + +	+	+ + +	+	+ + +	+	No
CD69^+^ T cells	+	+	+	−	+	=	+	−	No
CD69^+^ NK cells	+ +	+	+	=	+ +	+	+ + +	=	Yes
CD69^+^ B cells	+ +	+	+	+	+ + +	−	−	+	Yes
T_reg_ cells	=	=	+	−	+	+	+ +	=	No
Activated T_reg_ cells^a^	=	=	+	=	+ +	=	+ + +	+	No

Arm 1: zolbetuximab + ZA; Arm 2: zolbetuximab + ZA + IL-2 (1 mIU); Arm 3: zolbetuximab + ZA + IL-2 (3 mIU); Arm 4: zolbetuximab.  +  +  + Change rate: ≥ 3.0-fold; +  + Change rate: ≥ 2.0-fold and < 3.0-fold; + Change rate: > 1.2-fold and < 2.0-fold; = Change rate: ≤ 1.2-fold and ≥ 0.8-fold; − Change rate: < 0.8-fold and ≥ 0.5-fold; − − Change rate: < 0.5-fold

*D* days posttreatment initiation, *IL-2* interleukin 2, *NK* natural killer, *T*_*reg*_ regulatory T cell, *ZA* zoledronic acid

^a^Activation marker: FoxP3highCD45RA^−^

### Effects of ZA and IL-2 on γ9δ2 T cells

**Fig. 2:**
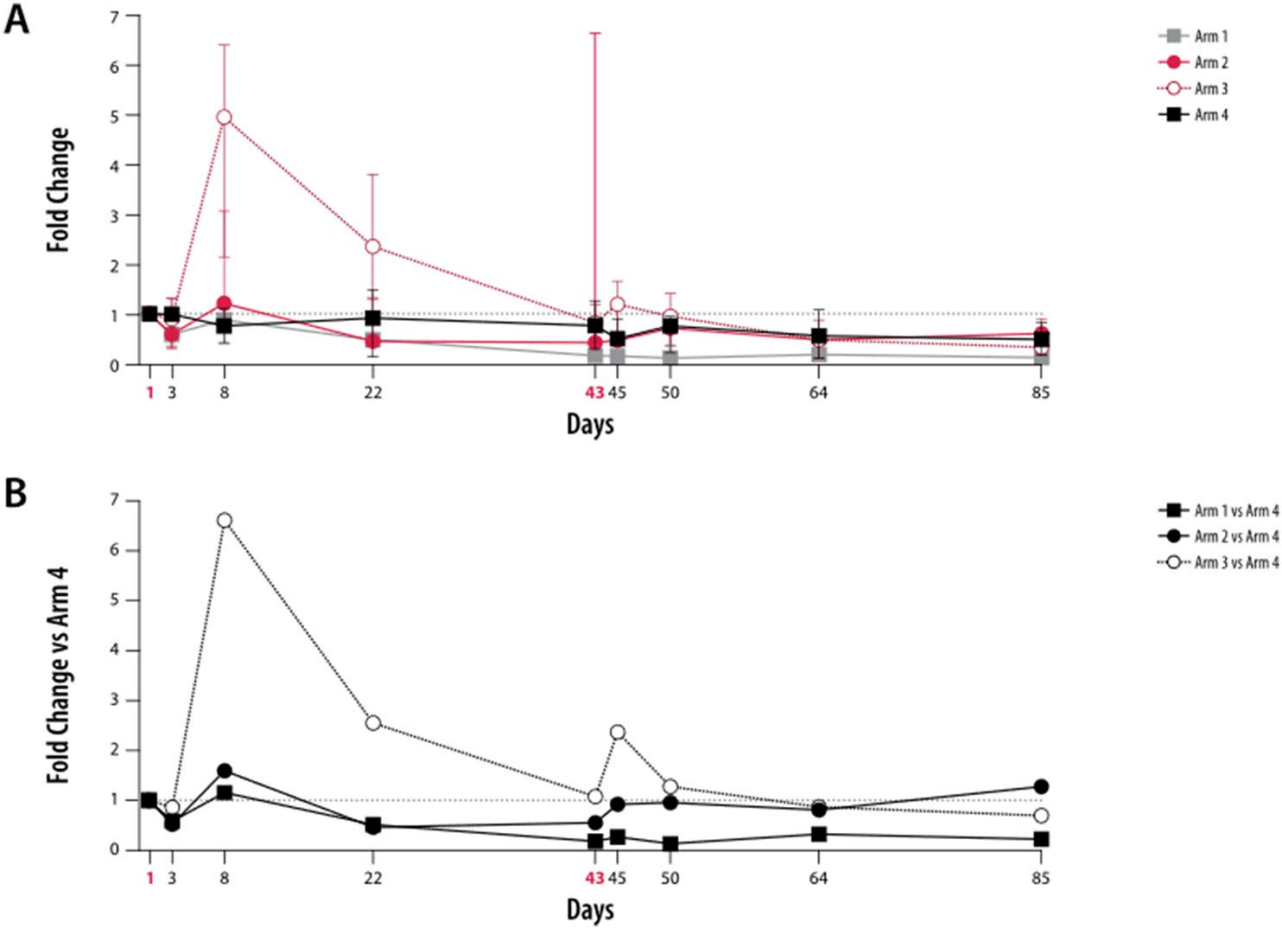
Kinetic profile of γ9δ2 T-cell frequency in lymphocytes. Kinetic profile of γ9δ2 T-cell frequency in lymphocytes. Peripheral blood mononuclear cells from patients were analyzed by flow cytometry at nine different time points (D1, D3, D8, D22, D43, D45, D50, D64, and D85). **a** Fold changes of median γ9δ2 T-cell frequencies (Vd2^+^CD3^+^ in lymphocytes) relative to pretreatment (D1). Arms 2 and 3 are shown in red; the lower error bar represents Q1 and the upper error bar Q3. **b** Median ratios in (**b**) were normalized to the median ratios of the control arm on the respective days. Red, bold font indicates the ZA and ZA + IL-2 treatment days. Table lists medians of Arms 1 to 3 vs. Arm 4 (control). Error bars represent mean ± SD. Arm 1: zolbetuximab + ZA; Arm 2: zolbetuximab + ZA + IL-2 (1 mIU); Arm 3: zolbetuximab + ZA + IL-2 (3 mIU); Arm 4: zolbetuximab. *D* Day, *IL-2* interleukin 2, *mIU* million international units, *SD* standard deviation, *ZA* zoledronic acid

Patients treated with zolbetuximab + ZA + intermediate-dose IL-2 (Arm 3) experienced expansion of γ9δ2 T cells by Day 8. A fivefold increase in median γ9δ2 T-cell frequency was observed by Day 8, compared with pretreatment levels (Fig. 2a), and a 6.6-fold increase was observed compared with zolbetuximab alone (Fig. 2b). Patients treated with zolbetuximab + ZA (Arm 1) or zolbetuximab + low-dose IL-2 (Arm 2) had minimal increases in median γ9δ2 T-cell frequency compared with zolbetuximab alone at Day 8. During the long-term (Days 1–85) timeframe, a steady decrease versus baseline in median γ9δ2 T lymphocytes was observed in all arms.

**Fig. 3:**
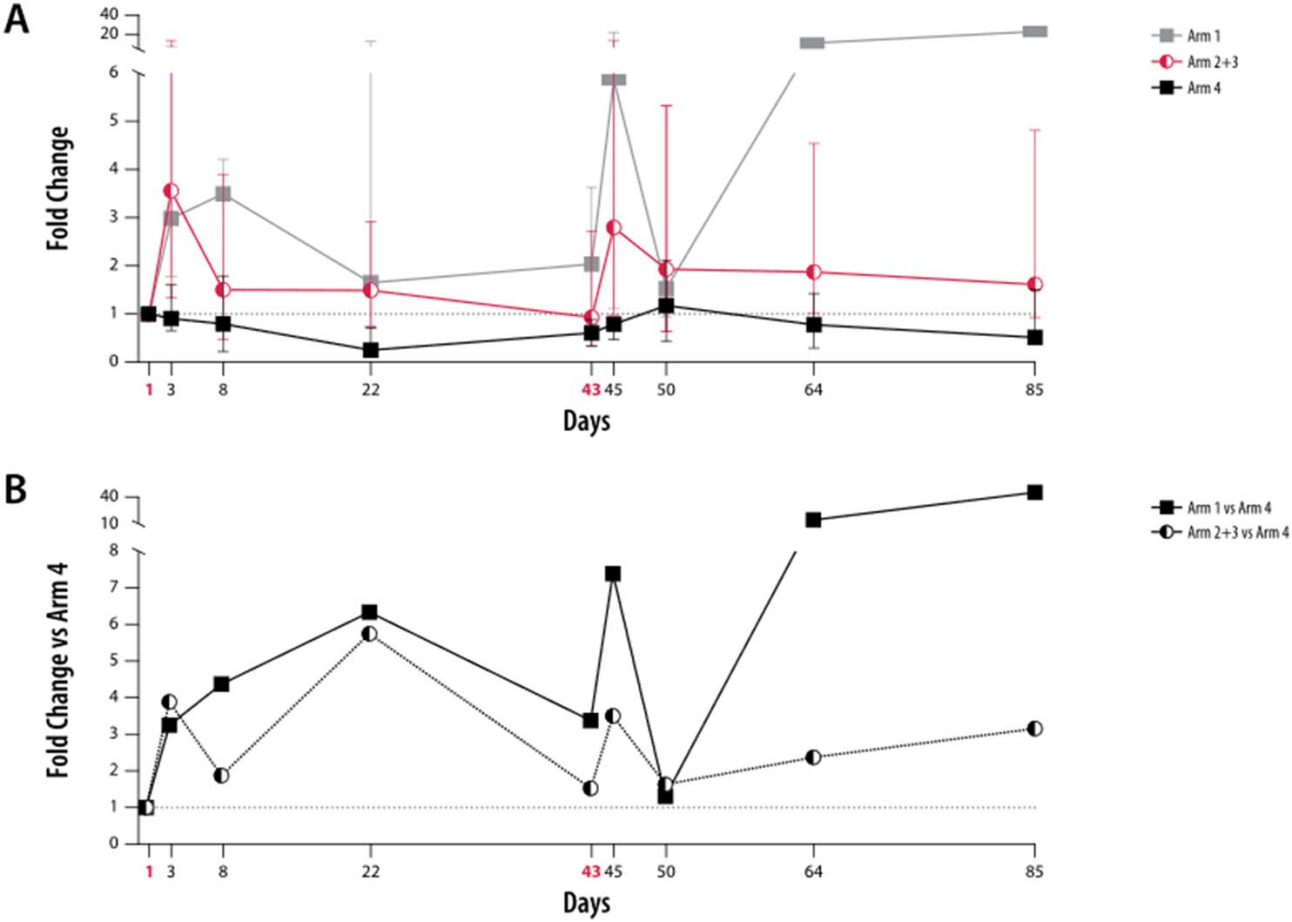
Kinetic profile of activated CD69^+^ γ9δ2 T cells in lymphocytes. Peripheral blood mononuclear cells from patients were analyzed by flow cytometry at nine different time points (D1, D3, D8, D22, D43, D45, D50, D64, and D85). **a** Fold changes of the median frequencies of activated CD69^+^Vd2^+^CD3^+^. **b** Median values normalized to the median values of the control arm on the respective days. Medians of the same treatment arm at different time points are connected and the red, bold font indicates the ZA and ZA + IL-2 treatment days. Error bars represent mean ± SD. Arm 1: zolbetuximab + ZA; Arm 2: zolbetuximab + ZA + IL-2 (1 mIU); Arm 3: zolbetuximab + ZA + IL-2 (3 mIU); Arm 4: zolbetuximab. *D* Day, *IL-2* interleukin 2, *mIU* million international units, *SD* standard deviation, *ZA* zoledronic acid

Frequencies of activated CD69^+^ γ9δ2 T cells increased from pretreatment levels in all zolbetuximab + ZA-treated patients (Arms 1, 2, and 3) from Day 3 onward and remained above pretreatment levels until Day 85 (Fig. 3). No increase in activated CD69^+^ γ9δ2 T-cell frequency was observed in patients treated with zolbetuximab alone. After the second zolbetuximab + ZA + IL-2 treatment in Cycle 3, an increase in the frequency of activated CD69^+^ γ9δ2 T cells was again observed in all ZA-treated patients. Although increases in activated CD69^+^ γ9δ2 T-cell frequencies in all ZA-treated patients were of similar magnitude, increases were slightly higher in patients who did not receive IL-2 than in those who received IL-2.

### Effects of ZA and IL-2 on NK cells

**Fig. 4:**
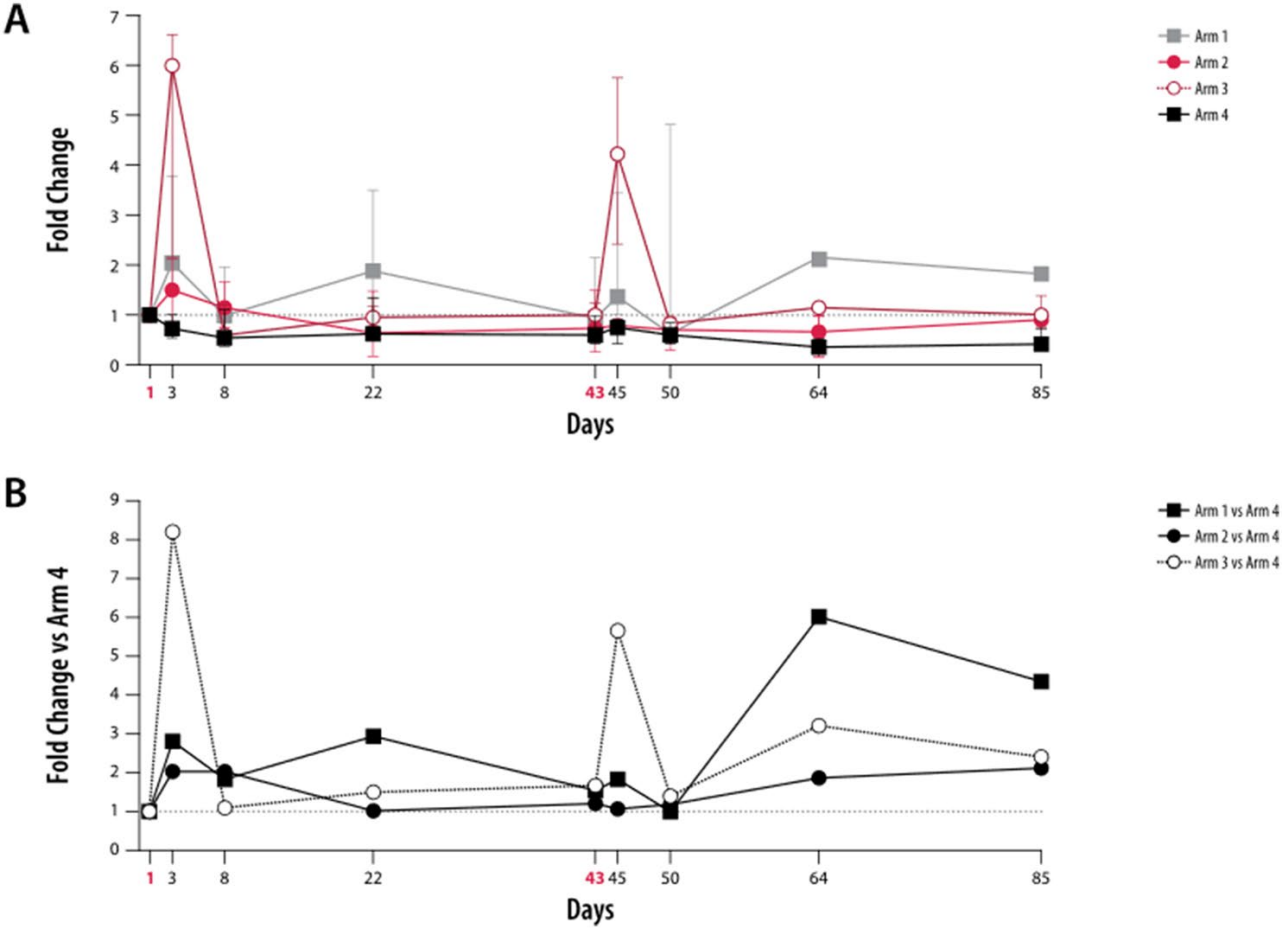
Kinetic profile of activated CD69^+^ NK cells. Peripheral blood mononuclear cells from patients were analyzed by flow cytometry at nine different time points (D1, D3, D8, D22, D43, D45, D50, D64, and D85). **a** Median frequencies relative to pretreatment (D1) of activated NK cells (CD69^+^ in CD16^+^CD56^+^). Arms 2 and 3 are shown in red; the lower error bar represents Q1 and the upper error bar Q3. **b** Median ratios shown in (**a**) were normalized to the median ratios of control Arm 4 on respective days. Red, bold font indicates the ZA and ZA + IL-2 treatment days. Error bars represent mean ± SD. Arm 1: zolbetuximab + ZA; Arm 2: zolbetuximab + ZA + IL-2 (1 mIU); Arm 3: zolbetuximab + ZA + IL-2 (3 mIU); Arm 4: zolbetuximab. *D* Day, *IL-2* interleukin 2, *mIU* million international units, *NK* natural killer, *SD* standard deviation, *ZA* zoledronic acid

There was no difference observed in median NK cell frequency over time in patients treated with zolbetuximab + ZA and intermediate-dose or low-dose IL-2 versus those treated with zolbetuximab alone (Table 2). Patients treated with intermediate-dose IL-2 had an early increase in the median frequency of CD69^+^-activated NK cells within the lymphocyte population, occurring within 3 days after administration of ZA and IL-2 in both treatment cycles. This increase was short-lived, with activated CD69^+^ NK cell frequency returning to pretreatment levels by Day 8 in both treatment cycles (Fig. 4a). In patients who received ZA + intermediate-dose IL-2, an eightfold increase in activated CD69^+^ NK cell frequency after the first zolbetuximab + ZA + IL-2 administration relative to zolbetuximab alone was observed; this increase was less pronounced (sixfold) after the second zolbetuximab + ZA + IL-2 treatment (Fig. 4b).

### Effects of ZA and IL-2 on T_reg_ cells

**Fig. 5:**
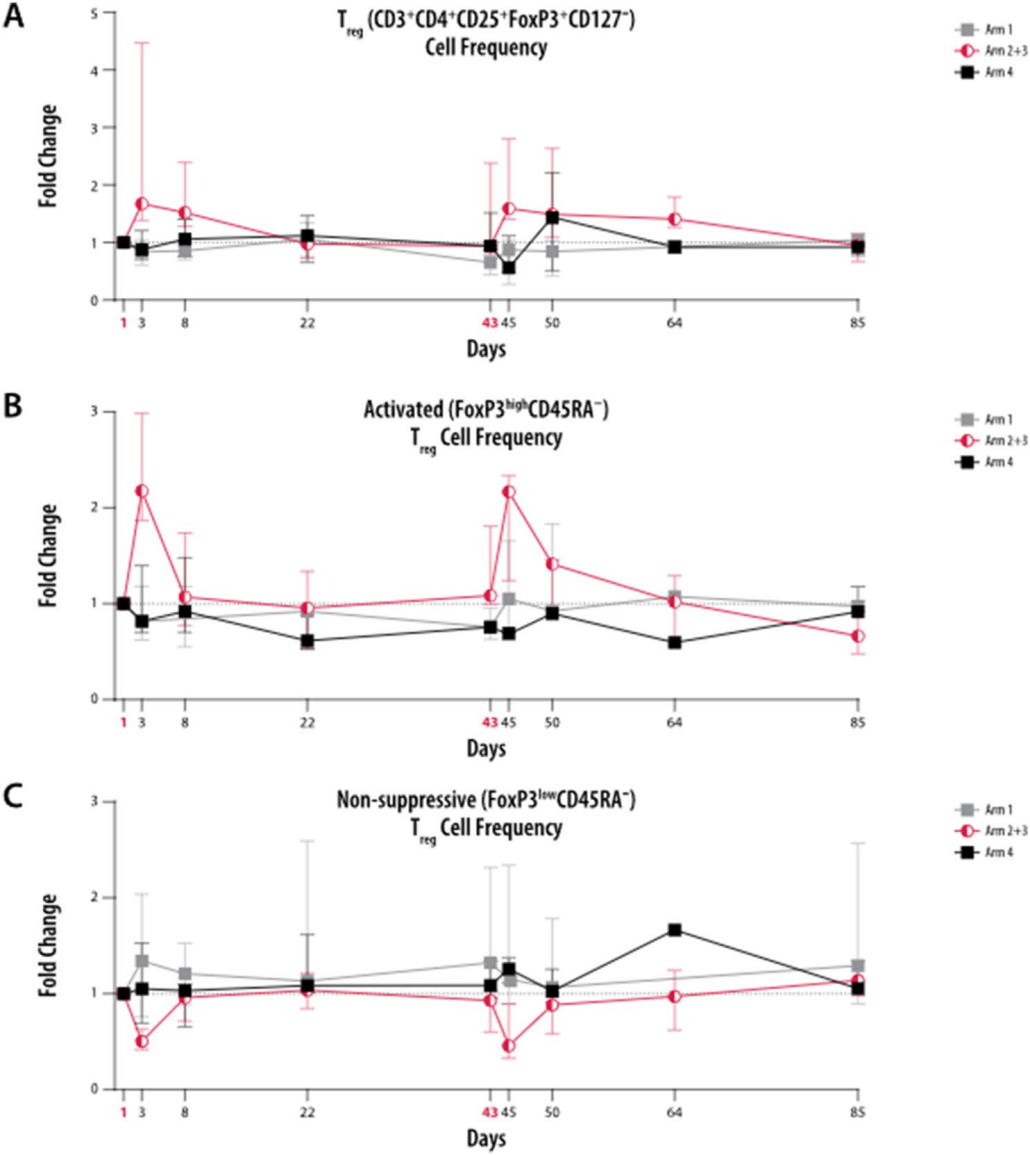
Kinetic profile of T_reg_ cell frequencies in T lymphocytes. Peripheral blood mononuclear cells from patients were analyzed by flow cytometry at nine different time points (D1, D3, D8, D22, D43, D45, D50, D64, and D85). Fold changes of the median frequencies of (CD3^+^CD4^+^CD25^+^FoxP3^+^CD127^–^) T_reg_ cells (**a**), activated (FoxP3^high^CD45RA^−^) T_reg_ cells (**b**), and non-suppressive (FoxP3^low^CD45RA^−^) T_reg_ cells (**c**) relative to respective medians of D1 are presented. Arms 2 and 3 are combined (red); the lower error bar represents Q1 and the upper error bar Q3. Red, bold font indicates ZA and ZA + IL-2 treatment days. Error bars represent mean ± SD. Arm 1: zolbetuximab + ZA; Arm 2: zolbetuximab + ZA + IL-2 (1 mIU); Arm 3: zolbetuximab + ZA + IL-2 (3 mIU); Arm 4: zolbetuximab. *D* Day, *IL-2* interleukin 2, *mIU* million international units, *SD* standard deviation, *ZA* zoledronic acid

The frequency of (CD3^+^CD4^+^CD25^+^FoxP3^+^CD127^−^) T_reg_ cells were upregulated 1.7-fold 2 days after treatment in patients who received both ZA and IL-2 (low- and intermediate-dose groups combined); this increase was maintained at near the same level (1.5-fold) up to Day 8, but subsequently declined to pretreatment levels by Days 22 and 43 (Fig. 5a). A similar increase (1.6-fold vs. baseline) in the frequency of (CD3^+^CD4^+^CD25^+^FoxP3^+^CD127^−^) T_reg_ cells was also noted 2 days after the second zolbetuximab + ZA + IL-2 treatment on Day 45 and was maintained at near the same level (1.4-fold) until Day 64 before declining to pretreatment levels by Day 85. There was little change in (CD3^+^CD4^+^CD25^+^FoxP3^+^CD127^−^) T_reg_ cell frequency in patients who received zolbetuximab + ZA or zolbetuximab alone, except for a 1.4-fold increase on Day 50 in the zolbetuximab arm.

In patients who received zolbetuximab + ZA + IL-2 (low-dose and intermediate-dose), the proportion of activated (FoxP3^high^CD45RA^−^) T_reg_ cells approximately doubled 2 days after the first treatment but returned to pretreatment levels by Day 8 (Fig. 5b). A similar twofold increase in activated (FoxP3^high^CD45RA^−^) T_reg_ cells was observed 2 days (Day 45) after the second zolbetuximab + ZA + IL-2 administration, however, the subsequent decline to pretreatment levels occurred over a longer duration than that observed after the first treatment. Changes in non-suppressive (FoxP3lowCD45RA^−^) T_reg_ cells were reciprocal to changes in activated (FoxP3highCD45RA^−^) T_reg_ cells, with a significant decline in zolbetuximab + ZA + IL-2-treated patients on Days 3 and 45 (Fig. 5c). There was little change over time in resting (FoxP3^low^CD45RA^+^) T_reg_ cells in patients who received zolbetuximab + ZA + IL-2, irrespective of IL-2 dose.

### Zolbetuximab-dependent ADCC activity

**Fig. 6:**
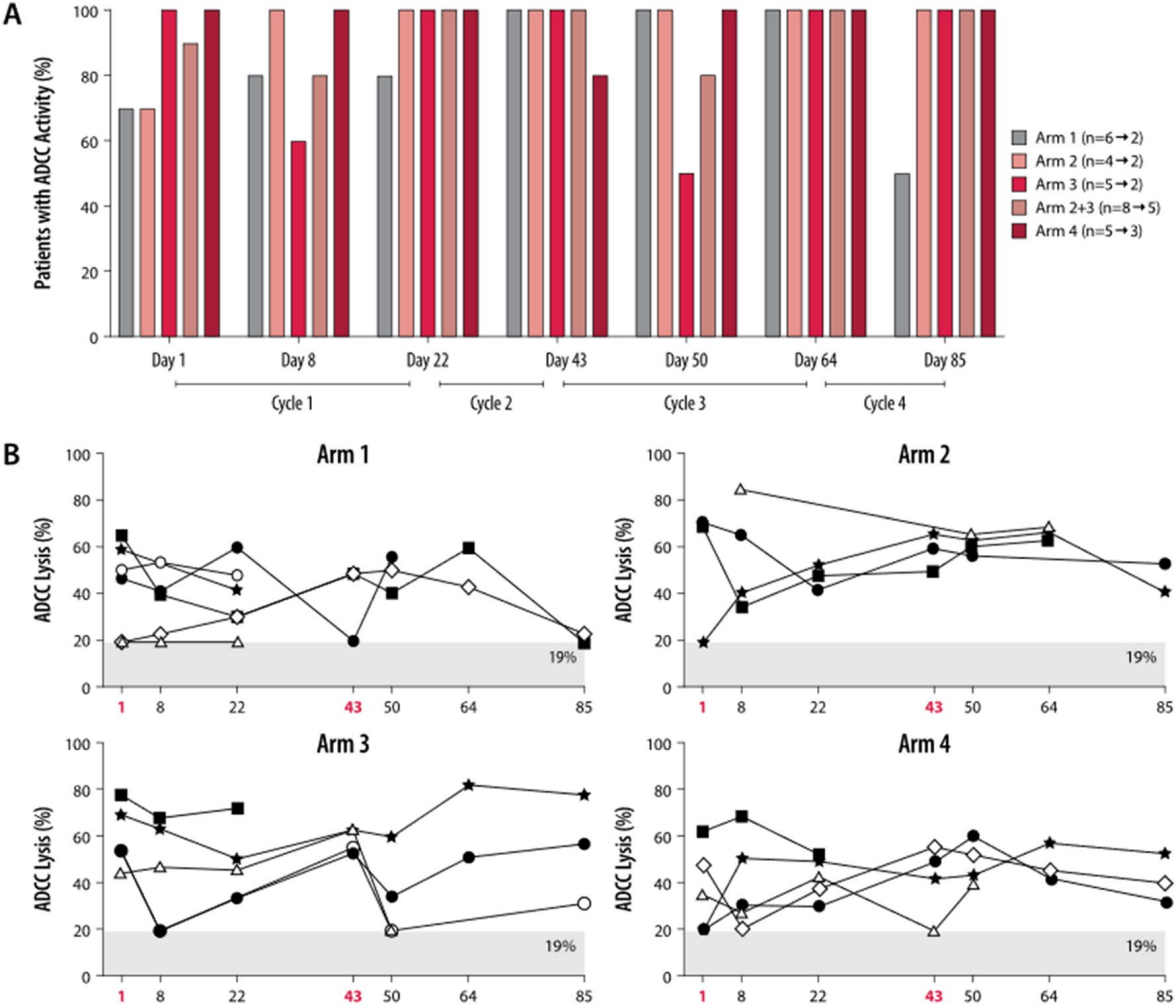
ADCC lysis activity during the course of treatment. Peripheral blood mononuclear cells were purified from patient blood samples and zolbetuximab-specific ADCC was determined using NUGC-4 target cells. **a** Proportion of patients with measurable ADCC-specific lysis (lysis > 19%) for all treatment arms on all study days until Day 85. Bracketed numbers indicate maximum to minimum patient numbers during the study for each treatment arm. **b** Corresponding patient values (symbols) grouped by treatment arm. Each line represents a single patient. The shaded area < 19% indicates the minimum threshold for specific lysis. Red, bold font indicates the ZA and ZA + IL-2 treatment days. Arm 1: zolbetuximab + ZA; Arm 2: zolbetuximab + ZA + IL-2 (1 mIU); Arm 3: zolbetuximab + ZA + IL-2 (3 mIU); Arm 4: zolbetuximab. *ADCC* antibody-dependent cell-mediated cytotoxicity, *IL-2* interleukin 2, *mIU* million international units, *ZA* zoledronic acid

Pretreatment median ADCC lysis activity was higher with zolbetuximab + ZA (48.3 [25.9; 56.8]) and zolbetuximab + ZA with intermediate-dose IL-2 (53.6 [46.0; 65.3]) or low-dose IL-2 (69.2 [44.1; 70.1]) than with zolbetuximab alone (34.6 [20.1; 47.3]). Most of the immune effector cell samples had measurable ADCC specific lysis from Day 1 through Day 85 (Fig. 6a), with maximum lysis ranging from 20% to 90% (Fig. 6b). In individual patients, ADCC activity during treatment fluctuated, but remained generally robust with only transient decreases (Fig. 6b; Table 3). In three of the four patients treated with intermediate-dose IL-2 who were evaluable for both zolbetuximab + ZA + IL-2 treatment cycles, ADCC lysis activity peaked at the time of ZA + IL-2 administration and decreased before the next ZA + IL-2 administration; this trend was not observed in patients treated with low-dose IL-2. By combining the time points for changes in ADCC-specific activity from the first and second ZA/IL-2 treatments, results became more robust due to a higher sample number. At the later time points (Day 8 and Day 50), most patients treated with zolbetuximab alone (56%) showed an increase in ADCC-specific activity after treatment (Table 3). Most patients treated with zolbetuximab + ZA + IL-2 lower dose (57%) and zolbetuximab + ZA + IL-2 any dose (58%) had decreased ADCC-specific activity after treatment at the later time points. At the delayed and late time points, the majority of patients (56–100%) did not show any qualitative change in ADCC-specific activity.

**Table 3:** Qualitative changes in ADCC activity over time across treatment arms

Change	Time point	Arm 1 % (#/total)	Arm 2 % (#/total)	Arm 3 % (#/total)	Arms 2 + 3 % (#/total)	Arm 4 % (#/total)
Increase	Higher (D8, D50)	17 (1/6)	20 (1/5)	0 (0/7)	8 (1/12)	56 (5/9)
	Delayed (D22, D64)	13 (1/8)	0 (0/4)	20 (1/5)	11 (1/9)	0 (0/8)
	Late (D43, D85)	0 (0/3)	0 (0/4)	14 (1/7)	9 (1/11)	0 (0/6)
Decrease	Higher	50 (3/6)	40 (2/5)	57 (4/7)	58 (7/12)	22 (2/9)
	Delayed	13 (1/8)	25 (1/4)	20 (1/5)	33 (3/9)	25 (2/8)
	Late	33 (1/3)	25 (1/4)	0 (0/7)	9 (1/11)	0 (0/6)
No change	Higher	33 (2/6)	40 (2/5)	43 (3/7)	33 (4/12)	22 (2/9)
	Delayed	75 (6/8)	75 (3/4)	60 (3/5)	56 (5/9)	75 (6/8)
	Late	66 (2/3)	75 (3/4)	86 (6/7)	82 (9 /11)	100 (6/6)

Arm 1: zolbetuximab + ZA; Arm 2: zolbetuximab + ZA + IL-2 (1 mIU); Arm 3: zolbetuximab + ZA + IL-2 (3 mIU); Arm 4: zolbetuximab. At the individual patient level, ADCC activity, monitored for up to 85 days during treatment, fluctuated but remained robust with only temporary decreases

*ADCC* antibody-dependent cell-mediated cytotoxicity, *D* days after treatment initiation, *IL-2* interleukin 2, *mIU* million international units, *ZA* zoledronic acid

### Safety and tolerability

The combination of zolbetuximab with ZA, with or without the addition of IL-2, was generally well tolerated. Overall, 92.9% of patients (*n* = 26/28) reported at least one AE of any grade; commonly reported AEs of any grade are shown in Supplementary Table 3. Adverse events determined to be related to zolbetuximab, ZA, or IL-2 were reported in 60.7% (*n* = 17/28), 21.7% (*n* = 5/23), and 56.3% (*n* = 9/16) of patients, respectively (Table 4).

**Table 4:** Incidence of drug-related TEAEs in ≥ 2 patients in any arm in the safety population

	Zolbetuximab (*n* = 28)	ZA (*n* = 23)	IL-2 (*n* = 16)
Any treatment-related AE, *n* (%)	17 (60.7)	5 (21.7)	9 (56.3)
Nausea	14 (50.0)	1 (4.3)	0
Vomiting	13 (46.4)	0	0
Fatigue	7 (25.0)	1 (4.3)	4 (25.0)
Decreased appetite	4 (14.3)	0	0
Upper abdominal pain	2 (7.1)	0	0
Dyspepsia	2 (7.1)	0	0
Asthenia	2 (7.1)	0	0
Chills	2 (7.1)	1 (4.3)	0
Cough	2 (7.1)	0	0
Hypertension	2 (7.1)	0	0
Pain	0	1 (4.3)	2 (12.5)
Pyrexia	0	0	4 (25.0)

Arm 1: zolbetuximab + ZA; Arm 2: zolbetuximab + ZA + IL-2 (1 mIU); Arm 3: zolbetuximab + ZA + IL-2 (3 mIU); Arm 4: zolbetuximab

*AE* adverse event, *IL-2* interleukin-2, *mIU* million international units, *TEAEs* treatment-emergent adverse events, *ZA* zoledronic acid

In the overall population, 35.7% of patients (*n* = 10/28) experienced AEs that led to discontinuation of their study drug regimens (Supplementary Table 4). One patient (Arm 2, zolbetuximab + ZA + low-dose IL-2) discontinued zolbetuximab because of a treatment-related AE (general deterioration of physical health) and two patients (one patient each in Arms 2 and 3) discontinued from the study because of AEs (deterioration of general physical health and grade 1 erythema). Seven patients (25%) died from AEs, none of which were deemed related to study medications. The presence of ADAs was evaluated in 17 of 28 patients, all of whom were negative for ADAs. Additional safety information is available in the Supplementary Table 4.

### Antitumor response

Efficacy results are provided in Supplementary Table 5. In the clinical efficacy population (*n* = 19), there were no complete or partial responses, 11 patients (58%) had stable disease and 8 patients (42%) had progressive disease. Median PFS was 37.3 weeks (95% CI, 9.0–42.1) with zolbetuximab alone, which was numerically longer than in the other treatment arms (7.1 weeks [5.4–40.0] to 12.7 weeks [6.0–27.0]). Median OS was 60.9 weeks (95% CI, 35.1–60.9) in patients who received zolbetuximab + ZA + intermediate-dose IL-2 (Arm 3), which was numerically longer than in the other treatment arms (10.9 weeks [7.7–40.0] to 37.3 weeks [11.0–42.1]).

## Discussion

Induction of ADCC has been shown to be an important mode of action of zolbetuximab (Tureci et al. 2019a). The antigen-specific antibody directs immune effector cells (NK cells, γ9δ2 T cells, monocytes/macrophages or neutrophil granulocytes) to antigen-expressing tumor cells and specifically mediates tumor cell killing. The objective of this study was to determine whether pretreated patients with advanced G/GEJ adenocarcinoma exert proficient ADCC and whether they further benefit from ZA and IL-2. Most of the patients in this study were able to robustly induce zolbetuximab-mediated ADCC at baseline, which is remarkable as all of the investigated individuals were heavily pretreated. Median ADCC activity in different treatment arms was divergent at baseline and showed variations between 35% and 69%.

Addition of the immune-modulating drugs ZA and IL-2 to zolbetuximab therapy resulted in only short-lived increases in γ9δ2 T cells, NK cells, and CD69^+^-activated γ9δ2 T and B cells. According to the expansion and/or activation of the immune-effector cell subset γ9δ2 T cells and NK cells in the zolbetuximab + ZA + IL-2 arms, one would expect selective augmentation of on-target effects of zolbetuximab in those treatment arms, such as temporal changes in ADCC lysis activity. However, comparison of posttreatment ADCC activity with baseline did not show augmented activity. Patients treated with zolbetuximab + ZA + low-dose IL-2 (Arm 2) or zolbetuximab + ZA + intermediate-dose IL-2 (Arm 3) showed decreases in ADCC activity. Only the patients treated with zolbetuximab alone consistently showed increases posttreatment. The interaction of the different immune cell subsets at the site of the tumor is not clear but might explain the lack of observed ADCC activity.

Zolbetuximab as a single agent has shown promising results in phase 1 (Sahin et al. 2018), phase 2a (Tureci et al. 2019b), and phase 2 (Sahin et al. 2021) studies as a treatment for advanced adenocarcinoma of the stomach, lower esophagus, or GEJ. Because zolbetuximab executes its mode of action by immune effector activation, a primary objective of this study was to determine the impact of immune-modulating drugs as co-treatment with zolbetuximab therapy on frequencies and activation status of relevant immune cell populations. The possible expansion of γ9δ2 T cells by ZA/IL-2 treatment was of particular interest as we have previously shown the importance of this immune cell subset as it pertains to zolbetuximab’s ability to induce ADCC (Tureci et al. 2019a).

The combination of zolbetuximab + ZA + IL-2 had a manageable safety profile and a best overall response of stable disease in 58% of patient. The lack of ADA reactivity with zolbetuximab in this study is consistent with the absence of ADA reactivity in G/GEJ cancer patients receiving a single dose of zolbetuximab over the dose range of 33–1000 mg/m^2^ in a phase 1 study (Sahin et al. 2018) or biweekly zolbetuximab at doses ranging from 1300 to 3600 mg/m^2^ in the phase 2a MONO study (Tureci et al. 2019b). ADA reactivity was observed in only two of 162 zolbetuximab-treated patients in the phase 2 FAST study of CLDN18.2-positive G/GEJ cancer patients who received zolbetuximab plus EOX versus EOX alone (Sahin et al. 2021).

There are a few limitations to this study. Although we observed numerically different median PFS and OS between the different treatment arms, this exploratory study was not adequately powered to compare clinical outcomes across treatment arms. We caution readers not to overinterpret this observation, particularly given that underlying reasons for the observed differences remain unclear on the basis of the available data. In retrospect, the patient numbers per group were too small given the interindividual variances, and the time points of analysis may not have been optimal. Although low patient numbers and the divergent baseline ADCC activity make interpretation difficult, T_reg_ cells were also increased in the zolbetuximab + ZA + IL-2 arms and results of earlier studies have shown that T_reg_ cells can interfere with ADCC (Pedroza-Pacheco et al. 2013). In addition, given that some effect was observed at the intermediate IL-2 dose, investigation of a higher dose and longer follow-up may be worthwhile. Furthermore, the IL-2 formulation used in this study (aldesleukin) has a poorer pharmacokinetics/pharmacodynamics profile than improved IL-2 formulations (Hartimath et al. 2018). Finally, without tumor biology information, such as Cancer Genome Atlas classification and HER2 or PD-L1 expression, assessment of these potential confounders was not possible.

Taken together, the results of this study show that even though patients were heavily pretreated, they clearly responded to the immune-modulating agents ZA/IL-2 in combination with zolbetuximab. Both expansion and activation of γ9δ2 T cells and activation of NK cells were initiated 2 days after treatment with ZA and IL-2 in combination with zolbetuximab. As the increase in ADCC lysis rate could only be studied first on Day 8, no final conclusion on a simultaneous increase in ADCC lysis rate in peripheral PBMCs on Day 3 could be drawn, as immune cells may have homed into the tumor tissue already. In order to investigate more closely whether activation and expansion of γ9δ2 T cells and activation of NK cells lead to improved antitumor activity of immune cells, future studies should include isochronal collection of immune cell and ADCC samples.

In conclusion, increases in ADCC activity observed with zolbetuximab alone support the beneficial role of zolbetuximab in priming effector cells for ADCC. While co-treatment with ZA or ZA + IL-2 did not enhance this effect, conclusive evaluation was not feasible due to the limitations described above. Zolbetuximab continues to be investigated as first-line treatment in two phase 3 trials in combination with either capecitabine/oxaliplatin (Xu et al. 2020) or mFOLFOX6 (Shitara et al. 2020) in claudin 18.2 + /HER2 − advanced or metastatic G/GEJ adenocarcinomas.

## Supplementary Information

Below is the link to the electronic supplementary material.

Supplementary file1 (DOCX 51 KB)

## Data Availability

Studies conducted with product indications or formulations that remain in development are assessed after study completion to determine if Individual Participant Data can be shared. The plan to share Individual Participant Data is based on the status of product approval or termination of the compound, in addition to other study specific criteria described on www.clinicalstudydatarequest.com under “Sponsor Specific Details for Astellas.”
