# Supplementary material for: Immunological effects and activity of multiple doses of zolbetuximab in combination with zoledronic acid and interleukin-2 in a phase 1 study in patients with advanced gastric and gastroesophageal junction cancer
Source: J Cancer Res Clin Oncol. 2023 Jan 6;149(9):5937–50. doi: 10.1007/s00432-022-04459-3 (PMC10356865; doi:10.1007/s00432-022-04459-3)
Supplement: Supplementary file 1 — Supplementary file1 (DOCX 51 KB) [file 432_2022_4459_MOESM1_ESM.docx]

**SUPPLEMENTARY INFORMATION**

**Immunological effects and activity of multiple doses of zolbetuximab in combination with zoledronic acid and interleukin-2 in a phase 1 study in patients with advanced gastric and gastroesophageal junction cancer**

Florian Lordick^1^, Peter Thuss-Patience^2^, Michael Bitzer^3^, Daniel Maurus^4*^, Ugur Sahin^5,6,7^, Özlem Türeci^4*,7^

^1^Department of Oncology, Gastroenterology, Hepatology, Pulmonology and Infectious Diseases, University Cancer Center Leipzig (UCCL), Leipzig University Medical Center, Leipzig, Germany; ^2^Department of Hematology, Oncology and Tumor Immunology, Charité – University Medicine Berlin, Campus Virchow-Klinikum, Berlin, Germany; ^3^Department of Internal Medicine I, University Hospital, Eberhard Karls University Tuebingen, Tuebingen, Germany; ^4^Ganymed Pharmaceuticals GmbH (formerly Ganymed Pharmaceuticals AG), Mainz, Germany; ^5^Translational Oncology at the University Medical Center of the Johannes Gutenberg University Mainz, Mainz, Germany; ^6^University Medical Center of the Johannes Gutenberg University Mainz, Mainz, Germany; ^7^Biopharmaceutical New Technologies (BioNTech) Corporation, Mainz, Germany

*The author is currently employed at Ci3-Cluster of Individualized Immune Intervention, Mainz, Germany

Email: [florian.lordick@medizin.uni-leipzig.de](mailto:florian.lordick@medizin.uni-leipzig.de)

**Supplementary Table 1. Inclusion and exclusion criteria**

| **Inclusion criteria** |
| --- |
| 1. Histologically confirmed adenocarcinoma of the stomach, the esophagus, or the gastroesophageal junction. 2. Inoperable locally advanced disease, resections with complete resection with no microscopic residual tumor (R0), microscopic residual disease at the resection margin (R1), or macroscopic residual disease at the resection margin (R2) outcome or metastatic disease. Patients had received standard therapy and were refractory or recurrent. 3. CLDN18.2 expression confirmed by immunohistochemistry in paraffin embedded tumor tissue sample. Any tumor with 3+ staining intensity was eligible for treatment and tumors with a staining intensity of 2+ were to show this in at least 40% of the tumor cells. 4. Measurable and/or non-measurable disease as defined according to RECIST v1.1. 5. Age ≥18 years. 6. Written informed consent. 7. Eastern Cooperative Oncology Group performance status 0 to 1. 8. Life expectancy >3 months. 9. Adequate cardiac function (LVEF >50%) without a recent (within 6 months) history of myocardial infarction and/or unstable or uncontrolled angina. 10. Adequate hepatic function; bilirubin <3 x upper limit of normal (ULN), aspartate aminotransferase and alanine aminotransferase <2.5 x ULN (5 x ULN if liver metastases were present). 11. Adequate renal function; creatinine <1.5 x ULN, glomerular filtration rate (GFR) ≥60 mL/minute calculated or measured. If the calculated GFR was <60 mL/minute, a measured GFR based on 24-hour urine was required. The measured GFR always took precedence over the calculated GFR. 12. Adequate hematological function; absolute neutrophil count ≥1.5 x 10^9^/L; white blood cell count ≥3.5 x 10^9^/L; platelets ≥100 x 10^9^/L; hemoglobin (Hb) ≥9 g/dL (could have been post-transfusion). 13. Women of childbearing potential (last menstruation <2 years prior to enrollment): negative pregnancy test (human chorionic gonadotropin [β-HCG]) at screening phase and used a highly effective method of contraception during the treatment phase and for 12 weeks after the last infusion of the study medication. 14. Male patients whose sexual partners were women of childbearing potential must have used condoms during the treatment phase and for 12 weeks after the last infusion of the study medication. 15. The partners of the patients must have also applied contraceptive methods. |
| **Exclusion criteria** |
| 1. Prior hypersensitivity reaction or intolerance to one of the compounds of the study treatment. 2. Radiotherapy within 4 weeks of start of study treatment (2-week interval was allowed if palliative radiotherapy was given to bone metastatic side peripherally and patient recovered from acute toxicity). 3. Other investigational agents or devices used concurrently or within 4 weeks prior to this study. 4. Known human immunodeficiency virus infection or known symptomatic hepatitis (A, B, and/or C). 5. Clinical symptoms of cerebral metastases. 6. Clinically significant (i.e. active) cardiac disease. History of myocardial infarction or hospitalization for congestive heart failure within 12 months of enrollment. 7. Other clinically significant disease or co-morbidity which may have adversely affected the safe delivery of treatment within this study including, but not limited to any of the following: ongoing or active infection that required parenteral antibiotics and/or uncontrolled hypertension. 8. Psychiatric illness or social situations that would preclude study compliance. 9. Pregnancy or breastfeeding. 10. Gastric bleeding within the last 2 weeks prior to Day 1 of Cycle 1 (patients with symptomatic peptic ulcer were allowed if there was no bleeding). 11. Concurrent systemic immunosuppressive therapy, in particular systemic corticoids were to be stopped 2 weeks prior to therapy (Day 1 of Cycle 1). Inhaled and topically-applied steroids were the exception and were allowed. Systemic steroids were to be avoided as long as the patient was being treated with study medication. 12. History of clinically relevant interstitial lung disease (e.g. pneumonitis or pulmonary fibrosis). 13. Evidence of dyspnea at rest. If pulmonary function tests were performed, forced expiratory volume in 1 second/forced vital capacity <60%. 14. Patients with a history of aspirin sensitive asthma. 15. Prior or current active autoimmune disease that required management with immunosuppression. This includes inflammatory bowel disease, systemic vasculitis, scleroderma, psoriasis, hemolytic anemia, immune-mediated thrombocytopenia, rheumatoid arthritis, systemic lupus erythematosus, Sjogren's syndrome, sarcoidosis, or other rheumatologic disease. Asthma and chronic obstructive pulmonary disease that did not require daily systemic corticosteroids were acceptable. 16. Any history of seizures given increased seizure risk with IL-2. Patients with seizure disorder could be enrolled if on anticonvulsants and well controlled. 17. Sinusoidal obstruction syndrome, formerly known as veno-occlusive disease, if present, should have been stable or improving. 18. Patients with allografts. 19. Patients treated with any bisphosphonate-based therapeutic for any indication during the previous year. 20. Patients with recent (within 6 weeks) or planned dental or jaw surgery (e.g., extraction, implants). 21. Current clinically significant active dental problems; dental or fixture trauma, or a current or prior diagnosis of osteonecrosis of the jaw, of exposed bone in the mouth, or of slow healing after dental procedures. 22. Hypocalcemia that required medication. Corrected (adjusted for serum albumin) serum calcium <8 mg/dL (2 mmol/L). |

**Supplementary Table 2.** **Interleukin-2 (IL-2) dose modifications**

| Dose delays of ±2 days were allowed. |  |
| --- | --- |
| The following recommendations for dosing with IL-2 were to be followed in case the following toxicities were noticed during subcutaneous administration of IL-2: | |
| Grade 1 toxicity events | - No altered dose recommendations with the exception of grade 1 neurologic toxicity |
| Grade 1 to 4 neurologic toxicity | - For grade 1 to 3 toxicity, treatment was to be discontinued until the event had resolved; treatment was then to be resumed at a 20% dose reduction - For grade 4 toxicity, discontinuation of treatment was to be considered |
| Any grade 2 (including cardiac function, renal, hepatic) toxicity | - Treatment was to be discontinued until the event returned to grade 1 - For cardiac, hepatic, or renal toxicities, the dose of IL-2 was to be reduced by 20% |
| Any grade 3 and 4 (including hypotension, arrhythmia) toxicities | - Treatment was to be discontinued until the event was resolved - A maximum delay of one treatment cycle could have been considered for the resolution of grade 3 and 4 events - For persistent hypotension, headache, arrhythmia, cardiac, hepatic, and renal toxicities the dose of IL-2 was to be reduced by 20% |
| Fever | - IL-2 could have been discontinued for 24 hours and then restarted at a 20% dose reduced level |
| Abnormal white blood cell counts | - The dose of IL-2 could have been reduced by 20% for the remaining duration of the treatment course |

**Supplementary Table 3**. **Treatment-emergent adverse events occurring in ≥10% of the overall population (safety population)**

|  | | **Treatment groupa** | | | | | | | | | | | | | | | | **Overall *N*=8** | |  |
| --- | --- | --- | --- | --- | --- | --- | --- | --- | --- | --- | --- | --- | --- | --- | --- | --- | --- | --- | --- | --- |
|  |  | **Arm 1**  ***n*=7** | | | | **Arm 2**  ***n*=9** | | | | **Arm 3**  ***n*=7** | | | | **Arm 4**  ***n*=5** | | | |  |  |  |
|  |  | ***n* (%)** | | **Events** | | ***n* (%)** | | **Events** | | ***n* (%)** | | **Events** | | ***n* (%)** | | **Events** | | ***n* (%)** | **Events** |  |
| **Any treatment-emergent adverse event** | | **7 (100)** | | **47** | | **9 (100)** | | **93** | | **6 (85.7)** | | **44** | | **4 (80.0)** | | **71** | | **26 (92.9)** | **285** |  |
| **General disorders and administration-site conditions** | | **5 (71.4)** | | **12** | | **8 (88.9)** | | **19** | | **5 (71.4)** | | **13** | | **4 (80.0)** | | **10** | | **22 (78.6)** | **54** |  |
| Fatigue | | 3 (42.9) | | 3 | | 4 (44.4) | | 5 | | 3 (42.9) | | 5 | | 2 (40.0) | | 3 | | 12 (42.9) | 16 |  |
| General physical health deterioration | | 2 (28.6) | | 4 | | 1 (11.1) | | 1 | | 1 (14.3) | | 1 | | 2 (40.0) | | 2 | | 6 (21.4) | 8 |  |
| Peripheral edema | | 1 (14.3) | | 1 | | 2 (22.2) | | 2 | | 1 (14.3) | | 1 | | 1 (20.0) | | 1 | | 5 (17.9) | 5 |  |
| Pyrexia | | 1 (14.3) | | 1 | | 3 (33.3) | | 6 | | 1 (14.3) | | 2 | | 0 | | 0 | | 5 (17.9) | 9 |  |
| **Gastrointestinal disorders** | | **4 (57.1)** | | **8** | | **8 (88.9)** | | **44** | | **5 (71.4)** | | **17** | | **4 (80.0)** | | **32** | | **21 (75.0)** | **101** |  |
| Nausea | | 2 (28.6) | | 2 | | 6 (66.7) | | 13 | | 4 (57.1) | | 5 | | 3 (60.0) | | 7 | | 15 (53.6) | 27 |  |
| Vomiting | | 1 (14.3) | | 1 | | 6 (66.7) | | 13 | | 4 (57.1) | | 6 | | 4 (80.0) | | 13 | | 15 (53.6) | 33 |  |
| Abdominal pain | | 0 | | 0 | | 2 (22.2) | | 2 | | 1 (14.3) | | 1 | | 1 (20.0) | | 2 | | 4 (14.3) | 5 |  |
| Constipation | | 1 (14.3) | | 1 | | 1 (11.1) | | 1 | | 2 (28.6) | | 2 | | 0 | | 0 | | 4 (14.3) | 4 |  |
| Upper abdominal pain | | 0 | | 0 | | 2 (22.2) | | 3 | | 0 | | 0 | | 1 (20.0) | | 1 | | 3 (10.7) | 4 |  |
| Dysphagia | | 0 | | 0 | | 2 (22.2) | | 2 | | 0 | | 0 | | 1 (20.0) | | 1 | | 3 (10.7) | 3 |  |
| **Investigations** | | **2 (28.6)** | | **3** | | **4 (44.4)** | | **7** | | **2 (28.6)** | | **2** | | **3 (60.0)** | | **13** | | **11 (39.3)** | **27** |  |
| Increased gamma-glutamyl transferase | | 1 (14.3) | | 1 | | 2 (22.2) | | 2 | | 0 | | 0 | | 2 (40.0) | | 2 | | 5 (17.9) | 5 |  |
| Decreased weight | | 0 | | 0 | | 1 (11.1) | | 1 | | 1 (14.3) | | 1 | | 2 (40.0) | | 4 | | 4 (14.3) | 6 |  |
| Increased blood alkaline phosphatase | | 0 | | 0 | | 2 (22.2) | | 2 | | 0 | | 0 | | 1 (20.0) | | 1 | | 3 (10.7) | 3 |  |
| Increased C-reactive protein | | 1 (14.3) | | 1 | | 1 (11.1) | | 1 | | 1 (14.3) | | 1 | | 0 | | 0 | | 3 (10.7) | 3 |  |
| **Blood and lymphatic system disorders** | | **3 (42.9)** | | **6** | | **4 (44.4)** | | **4** | | **2 (28.6)** | | **2** | | **1 (20.0)** | | **2** | | **10 (35.7)** | **14** |  |
| Anemia | | 3 (42.9) | | 6 | | 2 (22.2) | | 2 | | 2 (28.6) | | 2 | | 1 (20.0) | | 2 | | 8 (28.6) | 12 |  |
| **Metabolism and nutritional disorders** | | **1 (14.3)** | | **1** | | **2 (22.2)** | | **7** | | **4 (57.1)** | | **5** | | **2 (40.0)** | | **2** | | **9 (32.1)** | **15** |  |
| Decreased appetite | | 1 (14.3) | | 1 | | 2 (22.2) | | 4 | | 3 (42.9) | | 4 | | 1 (20.0) | | 1 | | 7 (25.0) | 10 |  |
| **Respiratory, thoracic, and mediastinal disorders** | | **2 (28.6)** | | **7** | | **3 (33.3)** | | **5** | | **3 (42.9)** | | **4** | | **1 (20.0)** | | **3** | | **9 (32.1)** | **19** |  |
| Dyspnea | | 1 (14.3) | | 1 | | 1 (11.1) | | 1 | | 3 (42.9) | | 4 | | 0 | | 0 | | 5 (17.9) | 6 |  |
|  | |  | |  | |  | |  | |  | |  | |  | |  | |  |  |  |
|  | |  | |  | |  | | **Treatment groupa** | | | |  | |  | |  | |  | |  |
|  | | **Arm 1**  ***n*=7** | | | | **Arm 2**  ***n*=9** | | | | **Arm 3**  ***n*=7** | | | | **Arm 4**  ***n*=5** | | | | **Overall**  ***N*=28** | | |
|  | | **n (%)** | | **Events** | | **n (%)** | | **Events** | | **n (%)** | | **Events** | | **n (%)** | | **Events** | | **n (%)** | | **Events** |
| Cough | | 1 (14.3) | | 1 | | 3 (33.3) | | 3 | | 0 | | 0 | | 0 | | 0 | | 4 (14.3) | | 4 |
| Dysphonia | | 2 (28.6) | | 2 | | 0 | | 0 | | 0 | | 0 | | 1 (20.0) | | 1 | | 3 (10.7) | | 3 |
| **Musculoskeletal and connective tissue disorders** | | **3 (42.9)** | | **6** | | **3 (33.3)** | | **4** | | **3 (42.9)** | | **4** | | **0** | | **0** | | **9 (32.1)** | | **14** |
| Back pain | | 3 (42.9) | | 4 | | 1 (11.1) | | 2 | | 1 (14.3) | | 2 | | 0 | | 0 | | 5 (17.9) | | 8 |
| **Neoplasms benign, malignant, and unspecified (including cysts and polyps)** | | **3 (42.9)** | | **3** | | **3 (33.3)** | | **3** | | **0** | | **0** | | **1 (20.0)** | | **2** | | **7 (25.0)** | | **8** |
| Tumor pain | | 0 | | 0 | | 2 (22.2) | | 2 | | 0 | | 0 | | 1 (20.0) | | 2 | | 3 (10.7) | | 4 |
| **Vascular disorders** | | **2 (28.6)** | | **2** | | **2 (22.2)** | | **2** | | **1 (14.3)** | | **1** | | **1 (20.0)** | | **1** | | **6 (21.4)** | | **6** |
| Hypertension | | 2 (28.6) | | 2 | | 1 (11.1) | | 1 | | 0 | | 0 | | 1 (20.0) | | 1 | | 4 (14.3) | | 4 |
| Arm 1: zolbetuximab + ZA; Arm 2: zolbetuximab + ZA+IL-2 (1 mIU); Arm 3: zolbetuximab+ZA+IL-2 (3 mIU); Arm 4: zolbetuximab.  ^a^Each patient counted appears only once in a category; events are a total number.  *IL-2* interleukin-2, *mIU* million international units, *ZA* zoledronic acid. | | | | | | | | | | | | | | | | | | | | |

**Supplementary Table 4. Overview of treatment-emergent adverse events (safety population)**

| **Treatment-emergent adverse event** | **Treatment groupa** | | | | **Overall *N*=28** |
| --- | --- | --- | --- | --- | --- |
|  | **Arm 1**  ***n*=7** | **Arm 2**  ***n*=9** | **Arm 3**  ***n*=7** | **Arm 4**  ***n*=5** |  |
|  | ***n* (%)** | ***n* (%)** | ***n* (%)** | ***n* (%)** | ***n* (%)** |
| Any | 7 (100) | 9 (100) | 6 (85.7) | 4 (80.0) | 26 (92.9) |
| Grade ≥3 | 5 (71.4) | 8 (88.9) | 3 (42.9) | 3 (60.0) | 19 (67.9) |
| Serious | 3 (42.9) | 5 (55.6) | 2 (28.6) | 3 (60.0) | 13 (46.4) |
| Drug-relatedb |  |  |  |  |  |
| Zolbetuximab-related | 3 (42.9) | 6 (66.7) | 4 (57.1) | 4 (80.0) | 17 (60.7) |
| ZA-related | 1 (14.3) | 3 (33.1) | 1 (14.3) | ― | 5 (21.7) |
| IL-2-related | ― | 6 (66.7) | 3 (42.9) | ― | 9 (56.3) |
| Leading to study drug withdrawal | 1 (14.3) | 5 (55.6) | 2 (28.6) | 2 (40.0) | 10 (35.7) |
| Zolbetuximab-related | 0 | 1 (11.1) | 0 | 0 | 1 (3.6) |
| ZA-related | 0 | 0 | 0 | ― | 0 |
| IL-2-related | ― | 0 | 0 | ― | 0 |
| Leading to study discontinuation | 0 | 1 (11.1) | 1 (14.3) | 0 | 2 (7.1) |
| Leading to death | 2 (28.6) | 3 (33.3) | 1 (14.3) | 1 (20.0) | 7 (25.0) |
| Arm 1: zolbetuximab + ZA; Arm 2: zolbetuximab + ZA+IL-2 (1 mIU); Arm 3: zolbetuximab+ZA+IL-2 (3 mIU); Arm 4: zolbetuximab.  ^a^Each patient counted appears only once in a category.  ^b^Drug-related refers to possibly or definitely related, as determined by the investigator when reporting the treatment-emergent adverse event.  *IL-2* interleukin-2, *mIU* million international units, *ZA* zoledronic acid. | | | | | |

**Supplementary Table 5. Summary of tumor response and survival (clinical efficacy population)**

|  | **Treatment group** | | | | **Overall  *N*=19** |
| --- | --- | --- | --- | --- | --- |
|  | **Arm 1** ***n*=5** | **Arm 2** ***n*=5** | **Arm 3** ***n*=5** | **Arm 4** ***n*=4** |  |
| **Best confirmed response, n (%)** |  |  |  |  |  |
| Complete response | 0 | 0 | 0 | 0 | 0 |
| Progressive disease | 3 (60.0) | 2 (40.0) | 2 (40.0) | 1 (25.0) | 8 (42.1) |
| Partial response | 0 | 0 | 0 | 0 | 0 |
| Stable disease | 2 (40.0) | 3 (60.0) | 3 (60.0) | 3 (75.0) | 11 (57.9) |
| Not assessed | 0 | 0 | 0 | 0 | 0 |
| Not evaluable | 0 | 0 | 0 | 0 | 0 |
| Objective response rate | 0 | 0 | 0 | 0 | 0 |
| Disease control rate | 2 (40.0) | 3 (60.0) | 3 (60.0) | 3 (75.0) | 11 (57.9) |
| **Median (95% CI) progression-free survival (weeks)** | 7.1  (5.4-40.0) | 12.7  (6.0-27.0) | 7.3  (6.3-21.0) | 37.3  (9.0-42.1) | ― |
| **Median (95% CI) overall survival (weeks)** | 10.9  (7.7-40.0) | 25.9  (7.1-74.3) | 60.9  (35.1-60.9) | 37.3  (11.0-42.1) | ― |
| Arm 1: zolbetuximab + ZA; Arm 2: zolbetuximab + ZA+IL-2 (1 mIU); Arm 3: zolbetuximab+ZA+IL-2 (3 mIU); Arm 4: zolbetuximab.  Objective response rate = complete response + partial response. Disease control rate = complete response + partial response + stable disease.  *CI* confidence interval, *IL-2* interleukin-2, *mIU* million international units, *ZA* zoledronic acid. | | | | | |
